# Assessment of Surface Roughness of Composite Resins Polished With Different Systems and Speeds: A Comparative In Vitro Study

**DOI:** 10.7759/cureus.97277

**Published:** 2025-11-19

**Authors:** Anupma Solanki, Rishi Manan, Charu Dayal, Mahesh Mohan, Ankit Gupta, Nadeem Ashiq, Seema Gupta

**Affiliations:** 1 Department of Conservative Dentistry and Endodontics, Institute of Dental Studies and Technologies, Modinagar, IND; 2 Department of Orthodontics, Kothiwal Dental College and Research Centre, Moradabad, IND

**Keywords:** composite resin, in-vitro study, polishing, speed, surface roughness

## Abstract

Introduction: The surface smoothness of resin composite restorations is critical for their esthetic appeal and clinical longevity because rough surfaces may promote bacterial adhesion and compromise durability. This study aimed to compare the surface roughness of nanofilled resin composite restorations polished with Enhance, Sof-Lex Diamond, and Shape Guard systems at high and low speeds using Mylar strip-finished samples as a control. The objectives were to evaluate intergroup differences in surface roughness via profilometry, assess the impact of polishing speed, and identify the most effective system for achieving minimal roughness to enhance clinical outcomes.

Materials and methods: Seventy cylindrical composite discs (8 mm diameter × 4 mm depth) were fabricated using a nanofilled resin composite, polymerized with a curing unit, and stored in distilled water at 37°C for 24 h. Ten samples cured against Mylar strips served as the control group (Group 4) without further polishing. The remaining 60 samples were roughened with 600-grit sandpaper and randomly assigned to three groups (20 samples each): Group 1 (Enhance), Group 2 (Sof-Lex Diamond), and Group 3 (Shape Guard). Each group was subdivided into high-speed (15,000 revolutions per minute (rpm)) and low-speed (5,000 rpm) subgroups (10 samples each). Polishing was performed for 20 s under dry conditions using a micromotor with a contra-angle hand piece. The surface roughness (Ra, µm) was measured using a calibrated stylus profilometer, using three readings per sample to calculate the mean values. Data were analyzed using one-way analysis of variance (ANOVA) and Tukey’s post hoc test (p < 0.05).

Results: The Mylar control group exhibited the lowest mean surface roughness (0.21 ± 0.07 µm). Among polished groups, Sof-Lex at high speed produced the smoothest surface (0.23 ± 0.07 µm), followed by Shape Guard at high speed (0.27 ± 0.09 µm). The Enhance system at low speed yielded the highest surface roughness (0.42 ± 0.14 µm). ANOVA confirmed significant differences among the groups (p = 0.001), thus rejecting the null hypothesis. Sof-Lex and Shape Guard at high speed matched the smoothness of the control, whereas Enhance performed inferiorly.

Conclusion: The Sof-Lex Diamond and Shape Guard systems, particularly when utilized at elevated velocities, achieved superior surface smoothness that closely competed with Mylar-finished controls. These systems are recommended for clinical use to optimize restoration esthetics and durability, although further in vivo studies are needed to confirm their long-term performance.

## Introduction

Resin composite restorations have revolutionized modern dentistry by offering superior esthetic outcomes and functional durability that closely mimic the natural tooth structure. Their widespread adoption stems from advancements in material science, enabling seamless integration with enamel and dentin while addressing patient demands for minimally invasive tooth-colored restorations [1]. However, the long-term success of these restorations depends on multiple factors, including the intrinsic properties of the composite material, such as monomer composition, filler type, size, and distribution, and extrinsic clinical techniques, such as incremental placement, polymerization, and crucially surface finishing and polishing [2]. Inadequate polishing can lead to increased surface roughness (SR), which promotes plaque accumulation, gingival irritation, secondary caries, staining, and accelerated wear, ultimately compromising both esthetics and longevity [3]. Conversely, optimal polishing enhances smoothness, gloss, and resistance to oral challenges, thereby improving patient comfort and restoration performance.

Composite resins are broadly classified into microfilled, microhybrid, and nanocomposites, with the latter incorporating nanotechnology to enhance mechanical strength and polishability [4]. Nanofilled composites feature particles ranging from 0.1 to 100 nm, providing a balance of opacity, strength, and esthetics suitable for anterior and posterior applications [5]. Despite these advancements, achieving a clinically ideal surface remains a challenge. The initial curing against a Mylar strip yields a resin-rich layer with low SR (approximately 0.12 µm), but this layer degrades in the oral environment, exposing filler particles and necessitating post-curing finishing [3]. Polishing systems vary in design and abrasives: one-step systems such as Enhance (aluminum oxide-impregnated) offer simplicity but may leave micro-irregularities [6]; multi-step options such as Sof-Lex Diamond (aluminum oxide and diamond particles) adapt to contours for superior gloss [7]; and flexible blade systems such as Shape Guard ensure uniform pressure distribution [8]. The speed of application (high or low) further influences abrasive efficiency, with higher speeds potentially enhancing frictional heat and resin flow for smoother outcomes, although this varies by system [9].

SR thresholds are critical: values above 0.2 µm facilitate bacterial adhesion, while above 0.3 µm affect tactile perception [10]. Evaluation methods, including profilometry and scanning electron microscopy, quantify these parameters and guide clinical protocols. Despite extensive research, comparative data on polishing systems at various speeds remain limited, particularly for nanocomposites.

The aim of this study was to comparatively evaluate the SR of resin composite restorations polished using Enhance, Sof-Lex Diamond, and Shape Guard systems at high and low speeds, with a Mylar strip as a control. The objectives included assessing intergroup differences in SR via profilometry, analyzing speed-dependent effects, and determining the most effective system for minimizing SR to enhance clinical outcomes. The null hypothesis for the study was that there would not be any significant difference between the groups.

## Materials and methods

This in vitro study was conducted at the Department of Conservative Dentistry and Endodontics, Institute of Dental Studies and Technologies, Modinagar, Ghaziabad, India, using advanced laboratory equipment for sample preparation, polishing, and surface analysis. The study spanned over two months, encompassing sample fabrication, stabilization, polishing, SR measurement, and data analysis, with sufficient time allocated to ensure procedural accuracy and material consistency. As an in vitro study involving no human or animal subjects, ethical approval was not required, adhering to the guidelines for non-clinical research outlined by institutional and international standards for dental material testing.

Eligibility criteria ensured that only defect-free samples (without visible cracks, porosity, or incomplete polymerization) were included and verified via visual inspection under 10× magnification and dimensional checks with digital calipers (accuracy ±0.01 mm). Noncompliant samples were replaced to maintain a sample size of 70. Randomization was achieved using a computer-generated sequence to assign samples to groups, thus minimizing selection bias.

The sample size was calculated using the G*Power software (version 3.1.9.7; Heinrich-Heine-Universität Düsseldorf, Düsseldorf, Germany), using an effect size of 0.45, derived from a previous study comparing the SR of a composite disc polished using Sof-Lex at multiple speeds [9]. A priori analysis (F test family for multiple groups) with an alpha error of 5% and a power of 80% and a minimum of 10 samples per group was required. The present study comprised six experimental groups (based on the material and polishing speed) and one control group, resulting in a total sample size of 70, including the control group.

Seventy cylindrical composite disc samples were prepared using a nanofilled resin composite (Filtek Z350 XT, 3M ESPE, St. Paul, MN, USA; Lot No. N828123) that was incrementally layered into a standardized cylindrical metal mold (8 mm diameter × 4 mm depth). Each increment was polymerized using a light-emitting diode (LED) curing unit (Bluephase N, Ivoclar Vivadent, Schaan, Liechtenstein) at 1,200 mW/cm² for 20 s, ensuring a uniform curing depth and minimizing polymerization shrinkage artifacts. To establish a baseline, 10 samples were cured against Mylar strips (Dentsply Sirona, Charlotte, NC, USA) to produce a smooth, resin-rich surface and were designated as the control group (Group 4), which underwent no further polishing. All samples were stored in distilled water at 37 ± 1^0^C for 24 h in a temperature-controlled incubator to simulate oral conditions and allow post-polymerization stabilization, following the protocols suggested by previous studies for composite material testing.

To replicate clinical finishing, the remaining 60 samples were uniformly roughened using 600-grit silicon carbide sandpaper (3M, St. Paul, MN, USA) under a standardized pressure for 15 s each, creating a consistent baseline surface texture. These samples were then randomly allocated into three experimental groups of 20 each. Randomization was achieved using a computer-generated random sequence (Random.org), with group allocations concealed in opaque, sequentially numbered envelopes opened immediately before polishing to prevent allocation bias.

Group 1 was polished with the Enhance system (Dentsply Sirona, Charlotte, NC, USA), Group 2 was polished with the Sof-Lex diamond polishing system (3M ESPE, St. Paul, MN, USA), and Group 3 was polished with the Shape Guard polishing system (Coltene, Altstätten, Switzerland). Each experimental group was further subdivided into two subgroups of 10 samples polished at either high speed (15,000 revolutions per minute (rpm)) or low speed (5,000 rpm) using a micromotor with a contra-angle handpiece (NSK, Tokyo, Japan), adhering to manufacturer-specified protocols for each system. Polishing was performed with light and intermittent pressure for 20 s per sample under dry conditions to prevent water-induced variability, and fresh polishing instruments were used for each subgroup to avoid abrasive wear. A standardized polishing pressure of approximately 2 N was applied using a digital force gauge (Model DS2-5N, IMADA Inc., Aichi, Japan) to maintain consistent contact force across all samples during polishing. For each system, a new polishing tip/disc was used after every five specimens to prevent abrasive wear (Table 1).

**Table 1:** Distribution of study samples in experimental groups. rpm: revolutions per minute.

Experimental groups (n = 20 each)	Polishing systems (Manufacturer)	Subgroups (n = 10 each)	Polishing speed	Polishing time	Pressure/Medium
Group 1	Enhance (Dentsply Sirona, Charlotte, NC, USA)	Subgroup 1A	High speed (15,000 rpm)	20 s	Light, intermittent/dry
		Subgroup 1B	Low speed (5,000 rpm)	20 s	Light, intermittent/dry
Group 2	Sof‑Lex diamond polishing system (3M ESPE, St. Paul, MN, USA)	Subgroup 2A	High speed (15,000 rpm)	20 s	Light, intermittent/dry
		Subgroup 2B	Low speed (5,000 rpm)	20 s	Light, intermittent/dry
Group 3	Shape Guard polishing system (Coltene, Altstätten, Switzerland)	Subgroup 3A	High speed (15,000 rpm)	20 s	Light, intermittent/dry
		Subgroup 3B	Low speed (5,000 rpm)	20 s	Light, intermittent/dry

The SR (Ra, in micrometers) was measured using a portable stylus profilometer (TR200, Beijing TIME High Technology Ltd., Beijing, China) calibrated before each session against a manufacturer-provided standard to ensure measurement accuracy within ±0.01 µm. Three readings were taken at different locations on each sample surface, and the mean Ra value was calculated to account for the potential surface heterogeneity. The profilometer’s stylus tip (5 µm radius) was moved at a constant speed of 0.5 mm/s over a 4-mm tracing length, for surface texture measurement. Instrument reliability was verified through a pilot test on five duplicate samples, yielding a coefficient of variation below 5% and confirming high reproducibility. All procedures were performed by a single trained operator to eliminate inter-operator variability, and the environmental conditions (temperature, 23 ± 20°C; humidity, 50% ± 5%) were controlled to ensure consistency. Prior to the main experiment, the operator underwent calibration by performing 10 trial polishing procedures on pilot samples, followed by intra-operator reliability assessment. The calibration yielded an intra-class correlation coefficient (ICC) of >0.9 for surface roughness readings, confirming procedural consistency.

Data were recorded and analyzed using statistical software (SPSS 25.0, IBM, Armonk, NY, USA), and the normality of the continuous SR data was confirmed using the Shapiro-Wilk test. Descriptive statistics are presented as the mean ± standard deviation. Differences in SR among material groups were assessed using one-way analysis of variance (ANOVA), followed by a post hoc Tukey’s test for pairwise comparisons. Statistical significance was set at p < 0.05, and the p-value was adjusted for multiple comparisons.

## Results

The descriptive analysis of SR revealed a distinct performance across the groups. The control group finished with Mylar (control group) exhibited the lowest mean SR (0.21 ± 0.07 µm). Among the polished groups, Sof-Lex high speed produced the smoothest surface (0.23 ± 0.07 µm), a result closely matched by Shape Guard high speed (0.27 ± 0.09 µm). In contrast, the Enhance system, particularly at low speed, yielded the highest mean SR values (0.42 ± 0.14 µm). The data suggest that a high-speed polishing protocol, especially with Sof-Lex, is the most effective for achieving a superior surface finish (Table 2).

**Table 2:** Descriptive analysis of study groups for mean surface roughness (Ra, in micrometers) at different speeds. Data are presented as mean and standard deviation (SD), whereas samples are presented as frequency (N) and percentage (%), where N denotes number of samples in each group. CI: confidence interval.

Material	N (%)	Minimum	Maximum	95% CI for mean	Mean ± SD
Enhance at low speed	10 (14%)	0.24	0.67	0.31-0.52	0.42 ± 0.14
Enhance at high speed	10 (14%)	0.24	0.48	0.32-0.45	0.38 ± 0.09
Sof-Lex at low speed	10 (14%)	0.24	0.44	0.29-0.38	0.34 ± 0.06
Sof-Lex at high speed	10 (14%)	0.14	0.38	0.18-0.28	0.23 ± 0.07
Shape Guard at low speed	10 (14%)	0.16	0.61	0.23-0.42	0.32 ± 0.13
Shape Guard at high speed	10 (14%)	0.11	0.39	0.21-0.34	0.27 ± 0.09
Control group with Mylar strip	10 (14%)	0.07	0.29	0.16-0.26	0.21 ± 0.07

One-way ANOVA revealed a statistically significant difference in the SR among the different material and polishing speed groups (p = 0.001). This significant p-value indicates that the type of finishing and polishing system used had a substantial and measurable effect on the resultant surface texture of the composite resin. Therefore, the null hypothesis was rejected as a statistically significant difference was observed between the groups (Table 3).

**Table 3:** Intergroup comparison of surface roughness (Ra, in micrometers) using one-way analysis of variance (ANOVA). df: degree of freedom. *p < 0.001: highly statistical significance using one-way ANOVA.

Measures	Sum of squares	df	Mean square	F stats	p-value
Material	0.34	6	0.06	5.97	0.001*
Residual	0.6	63	0.01		
Total	0.95	69			

At low speed, the Enhance group produced a significantly rougher surface than the Sof-Lex at high speed, Shape Guard at high speed, and control groups. Similarly, the Enhance at high speed produced a significantly rougher surface than both Sof-Lex at high speed and control groups. No significant difference was found between the two polishing speeds for any of the single-material systems. Critically, the Sof-Lex at high speed, Shape Guard at high speed, and control groups were statistically indistinguishable from one another. The inference is that while the Enhance system yields inferior smoothness, both the Sof-Lex and Shape Guard systems, particularly at high speeds, can achieve a surface finish comparable to the Mylar strip control, making them clinically excellent choices for achieving high-gloss composites (Table 4).

**Table 4:** Pairwise comparison of groups using post hoc analysis with Tukey’s test. *p < 0.05: statistical significance using post hoc Tukey’s test. CI: confidence interval.

Pairwise groups	Mean difference	T stats	p-value	95% CI lower limit	95% CI upper limit
Enhance at low speed - Enhance at high speed	0.03	0.74	0.989	-0.11	0.17
Enhance at low speed - Sof-Lex at low speed	0.08	1.8	0.552	-0.06	0.22
Enhance at low speed - Sof-Lex at high speed	0.18	4.18	0.002*	0.04	0.32
Enhance at low speed - Shape Guard at low speed	0.09	2.09	0.370	-0.05	0.23
Enhance at low speed - Shape Guard at high speed	0.14	3.23	0.031*	0.00	0.28
Enhance at low speed - Control group	0.20	4.65	0.001*	0.07	0.34
Enhance at high speed - Sof-Lex at low speed	0.05	1.06	0.937	-0.09	0.18
Enhance at high speed - Sof-Lex at high speed	0.15	3.44	0.017*	0.01	0.29
Enhance at high speed - Shape Guard at low speed	0.06	1.35	0.825	-0.08	0.20
Enhance at high speed - Shape Guard at high speed	0.11	2.49	0.180	-0.03	0.25
Enhance at high speed - Control group	0.17	3.91	0.004*	0.03	0.31
Sof-Lex at low speed - Sof-Lex at high speed	0.10	2.38	0.224	-0.03	0.24
Sof-Lex at low speed - Shape Guard at low speed	0.01	0.29	1	-0.13	0.15
Sof-Lex at low speed - Shape Guard at high speed	0.06	1.43	0.783	-0.08	0.20
Sof-Lex at low speed - Control group	0.12	2.85	0.081	-0.01	0.26
Sof-Lex at high speed - Shape Guard at low speed	-0.09	-2.09	0.372	-0.23	0.05
Sof-Lex at high speed - Shape Guard at high speed	-0.04	-0.95	0.963	-0.18	0.10
Sof-Lex at high speed - Control group	0.02	0.47	0.999	-0.12	0.16
Shape Guard at low speed - Shape Guard at high speed	0.05	1.14	0.913	-0.09	0.19
Shape Guard at low speed - Control group	0.11	2.56	0.155	-0.03	0.25
Shape Guard at high speed - Control group	0.06	1.42	0.788	-0.08	0.20

## Discussion

The findings of this study highlight the varying efficacies of different polishing systems in achieving optimal surface smoothness on nanofilled resin composites, underscoring the importance of system selection and operational parameters in restorative dentistry. The superior performance of Sof-Lex Diamond and Shape Guard systems, particularly at higher speeds, aligns with their multi-step abrasive designs that progressively refine the surface by removing larger filler particles and smoothing the resin matrix [3]. In contrast, the coarse aluminum oxide points of the Enhance system may leave residual scratches, leading to elevated SR values. This disparity can be attributed to the differences in abrasive composition and flexibility. Sof-Lex employs diamond-impregnated discs that adapt well to composite contours, whereas Shape Guard's silicone-based polishers incorporate finer abrasives for enhanced gloss and reduced micro-irregularities. The hardness of aluminum oxide is significantly higher than that of silicon dioxide.

A comparative evaluation by Chour et al. [11] demonstrated that Sof-Lex discs yielded significantly lower SR, which was attributed to its sequential grit progression that minimized surface defects. In an in vitro analysis by Reis et al. [12], it was found that one-step polishing with a Mylar strip produced the smoothest surface compared to multi-step polishing with Sof-lex. The results of our study also supported the same, where the lowest SR was found for the control group (polishing with the Mylar strip). A review by Teughels et al. [13] reported that polishing to a threshold of Ra < 0.2 μm can prevent the formation of a biofilm on dental materials. The mean surface roughness with control group was found to be 0.21 µm in our study. All other polishing systems produced rougher surfaces than the control group.

Barbosa et al. [14] reported the lowest SR values with Sof-lex (Ra: 0.11 to 0.25 μm). Similar results were reported in a systematic review, where multistep polishing with Sof-Lex discs was the most effective method of polishing composites [15]. These studies corroborate the current results, suggesting that the effectiveness stems from the ability of the systems to homogenize the filler-resin interface without excessive matrix removal, which could otherwise expose the filler particles and increase roughness.

Regarding the influence of polishing speed, the trend toward smoother surfaces at higher rpm (15,000 rpm) versus lower revolutions (5,000 rpm) may be related to enhanced abrasive efficiency and reduced heat generation at optimal speeds, preventing softening or smearing of the resin that could occur at suboptimal velocities. Higher speeds facilitate better particle removal and surface planarization, as the centrifugal force aids in the even distribution of pressure and abrasives. This is consistent with the findings of Chavali et al. [16], who reported that polishing at 15,000 rpm produced higher gloss and lower SR, compared to polishing at a lower speed (5,000 rpm), owing to improved cutting action and minimized vibrational artifacts. Similar findings have been reported in another study by Tepe et al. [9]. Similar trends were reported by Watanabe et al. [17] and Korkmaz et al. [18], who emphasized that higher speeds enhance the frictional efficiency and improve polishing by promoting better resin flow and abrasive action.

The LED curing parameters (1,200 mW/cm² for 20 seconds) were selected based on previous evidence [19]. The lack of significant speed-related differences within each system in the present study suggests that material-specific protocols may mitigate speed effects, although overall, high-speed applications appear advantageous for systems such as Sof-Lex and Shape Guard. These insights are vital because excessive SR from suboptimal polishing can compromise the integrity of the composite over time.

Clinically, minimizing SR is paramount for the longevity and esthetics of resin composite restorations. Smoother surfaces, as achieved by Sof-Lex and Shape Guard at high speeds, reduce bacterial adhesion and plaque accumulation, thereby reducing the risk of secondary caries and gingival inflammation. Research by Teughels et al. [13] established an SR threshold of 0.2 µm, below which plaque retention is markedly diminished, aligning with the near-control levels observed here. Furthermore, high-gloss finishes enhance optical properties, improving color stability and resistance to extrinsic staining from dietary factors such as coffee or tobacco, as evidenced by Bagheri et al. [20], who linked low Ra to prolonged esthetic performance in anterior restorations. In posterior applications, reduced SR mitigates wear from occlusal forces and extends restoration durability. Thus, clinicians should prioritize multi-step diamond or silicone-based systems over single-step alternatives, such as Enhance, especially in high-esthetic zones, to optimize patient outcomes and reduce recall frequency for repolishing. Integrating high-speed protocols can further streamline chairside procedures and balance efficiency with quality.

Despite these implications, this study has inherent limitations typical of in vitro designs. The controlled laboratory environment does not replicate intraoral dynamics such as salivary flow, thermal cycling, or masticatory stresses, which can alter surface degradation over time. Additionally, the use of flat disc samples may not fully mimic the irregular contours of actual restorations, potentially influencing polishing efficacy and measurement accuracy. The single-operator approach, while reducing variability, limits the generalizability to diverse clinical skill levels. Moreover, the dry polishing conditions excluded water coolant effects, which might have affected the heat buildup and surface integrity in vivo. Future studies should incorporate aging simulations, such as thermocycling or abrasion testing, to evaluate long-term performance and include scanning electron microscopy for qualitative surface analysis alongside profilometry. Multicenter clinical trials could validate these findings in real-world settings.

## Conclusions

This study demonstrated that the choice of polishing system and speed significantly influenced the SR of the nanofilled resin composite restorations. The Sof-Lex diamond and Shape Guard systems, particularly at higher speeds, achieved smoother surfaces comparable to the Mylar strip control, outperforming the Enhance system. These findings highlight the importance of selecting multi-step diamond- or silicone-based polishing systems to optimize clinical outcomes, including esthetics and longevity, by minimizing bacterial adhesion and wear. While high-speed protocols enhance efficiency, in vitro limitations suggest cautious application in clinical practice, warranting further research to validate long-term performance under intraoral conditions.
